# A retrospective comparison of Sun’s tip-flexible semirigid ureterorenoscopy, super-mini percutaneous nephrolithotomy and flexible ureteroscopy applied to treat upper urinary tract calculi

**DOI:** 10.1186/s12894-024-01412-z

**Published:** 2024-02-14

**Authors:** Xinkai Huang, Liang Zhong, Zhifeng Huang, Haibiao Lai

**Affiliations:** Department of Urology, Zhongshan Hospital of Traditional Chinese Medicine Affiliated to Guangzhou University of Traditional Chinese Medicine, Zhongshan, 528400 China

**Keywords:** Sun’s tip-flexible semirigid ureterorenoscopy, Super-mini percutaneous nephrolithotomy, Flexible ureteroscopy, Upper urinary tract calculi

## Abstract

**Background:**

This retrospective study was conducted to compare the safety and efficacy of Sun’s tip-flexible semirigid ureterorenoscopy (tf-URS), super-mini percutaneous nephrolithotomy (SMP) and flexible ureteroscopy (FURS) in treating upper urinary tract calculi, including upper ureteral or renal calculi.

**Methods:**

We included patients with upper ureteral calculi or renal calculi 1.0–2.0 cm in size, who underwent tf-URS, SMP or FURS, respectively. The indicators reflecting safety and efficacy were compared among the three surgical techniques.

**Results:**

SMP presented with higher single stone crushing success rate, but longer operation time and postoperative hospital stay, more blood loss, and higher postoperative pain score compared with FURS and tf-URS (*P* < 0.05). The hospitalization cost of tf-URS group was lower than that of SMP and FURS groups (*P* < 0.05). The incidence of postoperative fever in tf-URS group was significantly higher than that in SMP group (*P* < 0.05). No significant difference was found in mucosal injury, perirenal hematoma, and stone-free rate at 3 months after surgery (*P* > 0.05).

**Conclusions:**

tf-URS and FURS have the advantages in minimal invasion, hospitalization cost, patient comfort, and hospital stay while SMP has higher stone-free rate. These three surgical techniques are safe, reliable and complementary, which should be selected according to the actual situation.

## Introduction

For the treatment of upper ureteral calculi or renal calculi, urological surgeons have always sought surgical techniques with less invasion, faster recovery, fewer complications, and lower cost. Sun’s tip-flexible semirigid ureterorenoscopy (tf-URS), super-mini percutaneous nephrolithotomy (SMP), and flexible ureteroscopy (FURS) are the commonly used lithotripsy methods in Chinese clinical centers [1–3]. It is essential to carry out a systematic comparison among these surgical techniques and clarify their respective scope of application so as to maximize their advantages and avoid their disadvantages.

In developing tf-URS, Sun et al. creatively combined a tip-flexible rigid scope with a retractable sheath to make tf-URS simultaneously possess the functions of a rigid ureteroscope and flexible ureteroscope. tf-URS is characterized by short learning course, easy operation, higher safety and easy maintenance [4, 5]. Zeng et al. reported their experience using micro-percutaneous nephrolithotomy (Micro-PCNL) and gradually developed SMP, a modified PCNL, which combines a Y-type sheath with negative pressure suction. Compared to traditional PCNL, SMP adopts F13 super-mini access for lithotripsy, thereby significantly lowering the incidence of complications such as bleeding and allowing the treatment of calculi ≤ 2 cm [6]. This technique has created an era of tubeless PCNL by combining with negative pressure suction, and the removal of nephrostomy tube and double J stent [7]. Thanks to the rapid development of manufacturing technology, FURS has made significant progress in treating urinary calculi. Especially in combination with a smaller ureter access sheath and negative pressure suction device, it has also been applied to treat staghorn calculi ≥ 2 cm [8].

Our clinic, as the Pearl River West Coast Prevention and Control Center of China Urinary Calculi Alliance, has accumulated some experience in minimally invasive techniques. This study retrospectively analyzed the efficacy and safety of three surgical techniques applied in treating upper urinary tract calculi.

## Patients and methods

### Patients

This study included patients with upper urinary tract calculi who first visited Zhongshan Hospital of Traditional Chinese Medicine between January 2021 and September 2021 for tf-URS (*n* = 51), SMP (*n* = 46), and FURS (*n* = 55) groups, respectively. All surgical procedures were conducted by a single surgeon who had been fully trained in these three surgical procedures. A certain surgical procedure was selected according to the patient’s preference after being well informed. Both B-scan ultrasonography and CT examination confirmed the existence of calculi. The degree of hydronephrosis was evaluated by measuring the anteroposterior renal pelvis diameter using CT. The clinical data were provided by the “sample bank” of the hospital, and the patients were diagnosed based on the classification criteria of 2020 European Association of Urologists (EAU) and American Urological Association (AUA) guidelines [9]. The research was approved by the Institutional Review Board of the Ethics Committee of the hospital (No. 2020ZSZY-LLK-148). The patients were selected according to the inclusion criteria: (1) met the diagnostic standard for upper ureteral calculi (the upper ureter extends from the renal pelvis to the upper border of the sacrum) or renal calculi with a diameter of 1.0–2.0 cm; (2) free of obvious infection indication; (3) had a white blood cell count < 5/µl by urinalysis and a negative result of preoperative midstream urine culture; (4) 15–88 years of age; (5) underwent one of the three surgical techniques, follow-up and various tests. The excluded patients were those with major organ diseases such as heart and lung, severe liver and kidney dysfunction, history of mental illness, severe diabetes, structural malformation of urinary system, and overactivity of bladder.

### Tf-URS procedure

Patients in tf-URS group were not routinely pre-placed with double J stents, but if ureteral stenosis was found during surgery, the stents were then inserted and lithotripsy operation would be performed in the second-time surgery. We placed the patients in the lithotomy position and put tf-URS (Hangzhou Hawk Optical Electronic Instruments Co., Ltd., SN-V type ureterorenoscope, approval number: Z2014 No. 3,220,531) into the ureter under direct vision. After exploring the upper ureter or renal pelvis, we inserted a 220 μm holmium laser fiber (Shanghai Ruikeen Laser Technology Co., Ltd., approval number: Z2013 No. 3,240,216). Then we withdrew the rigid sheath by 15 cm and turned the scope end upwards up to 180 degrees and downwards up to 260 degrees, followed by probing and pulverizing calculi without using stone basket. Finally we checked the mucosal injury as slowly withdrawing the scope, and placed F5 double J stent without using the urethral catheter.

### SMP procedure

As a modified PCNL, SMP needed a lithotomy position in advance and an F5 ureteral catheter was inserted into the ureter followed by indwelling the urinary catheter. After changing to the prone position, we pre-positioned the puncture site under ultrasound guidance and performed the surgical disinfection steps. Next we re-positioned the puncture site, punctured the renal calyx’s fornix using a 16G needle, and inserted the zebra guidewire. Following channel dilation, we successively inserted F8 to F13 Y-type sheath along the guide wire, connected the negative pressure suction device and placed the YS261 super-mini nephroscope (Hangzhou Hawk Optical Electronic Instruments Co., Ltd., approval number: Z2010 No. 3,220,207). We inserted a 500 μm Ho:YAG laser (Shanghai Ruikeen Laser Technology Co., Ltd., approval number: Z2013 No. 3,240,216) and pulverized calculi, followed by negative pressure suction. Finally we indwelled F5 ureteral catheter connecting with the urethral catheter instead of a nephrostomy tube, which was removed based on CT examination at 72 h after surgery.

### FURS procedure

Patients in FURS group were not routinely pre-placed with double J stents, but if ureteral stenosis was found during surgery, the stents were then inserted and lithotripsy operation would be performed in the second-time surgery. We placed the patients in the lithotomy position and the Wolf F9/9.8 ureteroscope entered the ureter under the guidance of a zebra guidewire. After exploring the renal pelvis, we measured the distance between the external urethral orifice and renal pelvis, withdrew the ureteroscope and then left the zebra guidewire in the urinary tract system. According to the measured distance, we inserted F12 ureter access sheath to renal pelvis through the guidewire. Then we placed a 220 μm Ho: YAG holmium laser fiber, put F8.5 FURS (Hangzhou Hawk Optical Electronic Instruments Co., Ltd., SN-V type ureterorenoscope, approval number: Z2010 No. 3,223,175) into the integrated system through ureter access sheath, followed by probing and pulverizing calculi without using stone basket. Finally we checked the mucosal injury as slowly withdrawing the access sheath, and placed F5 double J stent without using the urethral catheter.

### Observation index

Operation time referred to a period from the time when the scope was inserted into the body to the time when the scope was removed from the body. For the definitions on stone-free status, different studies [10, 11] has some differences on the size of stone fragments. In this study, stone-free status referred to the scope smoothly reaching target calices and pulverizing calculi into small fragments less than 2 mm [11]. Bleeding volume was calculated based on hemoglobin drop after surgery [12]. Postoperative hospital stay depended on when the patients met discharge criteria, including clear mind, stable vital signs, stable indexes of blood biochemistry test, blood routine, and urine analysis, mild or no pain. The visual analog scale (VAS) was utilized to measure pain intensity (score 0, painless; score 1-3, mild pain; score 4–6, moderate pain; score 7–10, severe pain) [13]. The hospitalization cost consisted of current hospitalization cost, second-time surgery cost and charge for removal of double J stent. The complications included infective fever, mucosal injury, and perirenal hematoma.

### Statistical analysis

Statistic analysis was performed using SPSS 25.0 software. Normally distributed data were expressed as mean ± standard deviation (SD). The difference among the three groups was compared through single factor analysis of variance (ANOVA), and Fisher’s least significant difference (LSD) test was further adopted to compare between-group difference. Count data were analyzed by chi-square (**χ**^**2**^) test. The mean CT value (HU) of the stone was calculated based on the CT values of stone core, edge and middle area. A significant difference was identified when *P* values were less than 0.05.

## Results

### Clinical characteristics of the study population

Patients with upper ureteral calculi or renal calculi underwent tf-URS (*n* = 51), SMP (*n* = 46) or FURS (*n* = 55). The clinical characteristics of these patients were summarized in Table 1. There was no significant difference in baseline data including age, body mass index (BMI), gender, stone location, mean stone CT value, calculi diameter, hydronephrosis, and the course of disease (*P* > 0.05).

**Table 1:** The comparison of clinical characteristics among the three groups

Group		tf-URS group (*n* = 51)	SMP group (*n* = 46)	FURS group (*n* = 55)	***F(x***^2^) value	***P*** value
Age		41.4 ± 14.6	41.4 ± 15.6	42.4 ± 13.2	*F* = 0.088	0.916
Body mass index (BMI)		24.25 ± 3.81	24.13 ± 4.07	24.66 ± 3.71	*F* = 0.276	0.759
Gender					*x*^2^ = 1.419	0.492
Male		25(49%)	28(61%)	29(53%)		
Female		26(51%)	18(39%)	26(47%)		
Stone location					*x*^2^ = 9.246	0.509
Renal calculi	Upper calyx	4(7.8%)	2(4.3%)	7(12.7%)		
	Middle calyx	10(19.6%)	6(13.0%)	5(9.1%)		
	Lower calyx	2(3.9%)	6(13.0%)	2(3.6%)		
	Renal pelvis	4(7.8%)	3(6.5%)	3(5.5%)		
Upper ureteral calculi		24(47.1%)	24(52.2%)	30(54.5%)		
Multiple locations^*^		7(13.7%)	5(10.9%)	8(14.5%)		
Mean CT value (HU)		956 ± 231	1031 ± 270	991 ± 242	*F* = 1.144	0.321
Calculi diameter (cm)		1.3 ± 0.3	1.4 ± 0.3	1.4 ± 0.3	*F* = 0.667	0.515
Hydronephrosis^#^(cm)		1.7 ± 0.8	1.8 ± 1.1	1.9 ± 1.1	*F* = 0.440	0.645
The course of disease^†^ (d)		26.7 ± 15.3	26.9 ± 15.3	26.9 ± 13.8	*F* = 0.004	0.996

^*^Multiple locations refers to the stones existing in two or more locations (upper calyx, middle calyx, lower calyx, renal pelvis or upper ureteral calculi)

^#^Hydronephrosis refers to the dilatation of the renal pelvis and/or calyces

^†^The course of disease refers to the days calculated from the onset of symptoms or a positive imaging test without symptoms

### The comparison of intraoperative and postoperative indexes

The intraoperative and postoperative indexes were compared in Table 2. The single stone crushing success rate of SMP group (97.8%) was significantly higher than those of tf-URS group (78.4%) and FURS group (78.2%) (*P* < 0.05). In tf-URS group, the remaining 11(21.6%) cases underwent the insertion of double J stents and required the second-time surgery while 9.8%(5/51) cases had ureteral calculi obstruction who required ureteroscopic lithotripsy. In SMP group, one case changed to perform extracorporeal shock wave lithotripsy (ESWL) due to the puncture failure of SMP. In FURS group, the remaining 12(21.8%) cases underwent the insertion of double J stents and required the second-time surgery while 5.5%(3/55) cases had ureteral calculi obstruction who required ureteroscopic lithotripsy.

**Table 2:** The comparison of intraoperative and postoperative indexes among the three groups

Group	tf-URS group (*n* = 51)	SMP group (*n* = 46)	FURS group (*n* = 55)	***F(x***^2^) value	***P*** value	Between-group difference		
Operation time (min)	46.5 ± 15.6	106.4 ± 17.0	52.5 ± 16.2	*F* = 198.831	0.000	*P* = 0.000^#^	*P* = 0.059^†^	*P* = 0.000^§^
Single stone crushing success rate^*^	40(78.4%)	45(97.8%)	43(78.2%)	*x*^2^ = 9.198	0.010	*P* = 0.004^#^	*P* = 0.975^†^	*P* = 0.003^§^
Hemoglobin drop after surgery (g/L)	4.3 ± 2.1	14.3 ± 5.0	5.0 ± 3.2	*F* = 115.228	0.000	*P* = 0.000^#^	*P* = 0.322^†^	*P* = 0.000^§^
Postoperative hospital stay (d)	2.2 ± 1.1	4.7 ± 3.3	2.3 ± 1.0	*F* = 23.703	0.000	*P* = 0.000^#^	*P* = 0.769^†^	*P* = 0.000^§^
Pain degree (score) 6 h after surgery	2.1 ± 0.8	4.0 ± 1.8	2.3 ± 1.4	*F* = 26.486	0.000	*P* = 0.000^#^	*P* = 0.472^†^	*P* = 0.000^§^
Hospitalization cost (in RMB ×10^4^)	1.1 ± 0.5	2.0 ± 1.1	1.6 ± 0.6	*F* = 15.271	0.000	*P* = 0.000^#^	*P* = 0.002^†^	*P* = 0.011^§^
Stone-free rate at 3 months after surgery	43(84.3%)	44(95.7%)	47(85.5%)	*x*^2^ = 3.582	0.167			

^*^Single stone crushing success rate refers to the scope’s access to the collecting system followed by exploring and crushing stones successfully

^#^The difference between tf-URS group and SMP group

^†^The difference between tf-URS group and FURS group

^§^The difference between SMP group and FURS group

But SMP group had longer operation time and postoperative hospital stay, more blood loss, and higher postoperative pain score compared with FURS and tf-URS groups (*P* < 0.05). The hospitalization cost of tf-URS group was lower than that of SMP and FURS groups (*P* < 0.05). No significant difference was found among them (*P* > 0.05) in stone-free rates at 3 months after surgery with tf-URS group, SMP group, and FURS group being 84.3%, 95.7%, and 85.5%, respectively. After removing double J stent, 5 cases in tf-URS group suffered a ureteral obstruction during calculi expulsion and required a second surgery, while 3 cases in FURS group required a second surgery. But no cases required a second surgery in SMP group.

### The comparison of complications

As shown in Table 3, the incidence of postoperative fever in tf-URS group was significantly higher than that in SMP group (*P* < 0.05), but there was no significant difference between tf-URS group and FURS group, and between SMP group and FURS group (*P* > 0.05). All the 12 cases with postoperative fever recovered well without urosepsis following anti-infection treatment. As for mucosal injury and perirenal hematoma, no significant difference was found among the three groups (*P* > 0.05) although 7 cases in SMP group had perirenal hematoma after surgery. All the 9 cases with ureteral mucosa injury were identified as first-degree [14] and recovered spontaneously after indwelling double J stent for four weeks without surgical treatment. A total of 12 cases had perirenal hematoma ≤ 2 cm, which underwent self-absorption without arterial embolization. No urine leakage and severe complications such as delayed bleeding, ureter prolapse, and urosepsis occurred in all the three groups.

**Table 3:** The comparison of complications among the three groups

Group	tf-URS group (*n* = 51)	SMP group (*n* = 46)	FURS group (*n* = 55)	***x***^2^ value	***P*** value	Between-group difference		
Fever	8(15.7%)	1(2.2%)	3(5.5%)	5.979	0.043	*P* = 0.022^#^	*P* = 0.084^†^	*P* = 0.400^§^
Mucosal injury	3(5.9%)	1(2.2%)	5(9.1%)	2.030	0.389			
Perirenal hematoma	3(5.9%)	7(15.2%)	2(3.6%)	4.445	0.096			

^#^The difference between tf-URS group and SMP group

^†^The difference between tf-URS group and FURS group

^§^The difference between SMP group and FURS group

## Discussion

Currently PCNL and FURS are the common surgical methods used to treat upper urinary tract calculi, while tf-URS and SMP (a modified PCNL) were firstly carried out in China with relatively few literature reports. And there are no related literatures comparing the three surgical methods tf-URS, SMP or FURS. This study aimed to provide our evidence and experience to the clinical practice how to select tf-URS, SMP or FURS in treating upper urinary tract calculi. The results above demonstrated that these three surgical techniques had their respective characteristics, which would help to clarify their scope of application. In the aspect of operation time, tf-URS group and FURS group had an obvious advantage over SMP group because the preceding two groups utilized natural access and thereby avoided the steps of position rearrangement and renal puncture.

For single stone crushing success rate, tf-URS group and FURS group would be restricted under the circumstance of ureterostenosis since they entered the ureter through natural access [4, 8]. The body width of tf-URS was F13, and the sheath width of FURS group was F12 or F14. Patients in tf-URS group and FURS group achieved single stone crushing success rate of 78.4% and 78.2%, presenting with 11 cases and 12 cases with ureteral stenosis during surgery who required the insert of double J stent, respectively, both of which were significantly lower than 97.8% in SMP group. The blood loss in SMP group performed using artificial percutaneous renal access, was significantly higher than that of tf-URS group and FURS group utilizing natural access. A suspended surgery occurred in only one case in SMP group due to bleeding. In tf-URS group and FURS group, the urethral catheter did not need to be indwelled. In SMP group, the ureteral catheter and urethral catheter were removed 72 h after surgery to avoid the influence of ureteral edema [15]. Therefore, SMP group had longer postoperative hospital stay than tf-URS group and FURS group. But the patients in SMP group did not need to suffer the removal of double J stent. The pain degree of SMP group was significantly higher than other two groups due to the indwelled urethral catheter and artificial percutaneous renal access. Moreover, the pain from the incision and the stimulation from the drainage tube increased discomfort.

The hospitalization cost included the sum of current hospitalization cost, second-time surgery cost and charge for removal of double J stent. The hospitalization cost of tf-URS group was the lowest, and its reasons could be that tf-URS group did not involve the cost of disposable access sheath, disposable puncture frame and negative pressure suction device and had a shorter hospital stay. The hospitalization cost of the FURS group was significantly higher than that of tf-URS group since FURS group has to use disposable ureter access sheath, and the service life of a flexible ureteroscope generally ends up with only 50–60 uses and high maintenance cost [16]. Therefore, tf-URS group has an obvious advantage in terms of hospitalization cost. tf-URS group and FURS group spontaneously expelled the stones while SMP group adopted intraoperative suction of stones under negative pressure. Therefore, the stone-free rate of SMP group was higher than that of tf-URS group and FURS group. The stone-free rate of tf-URS group was lower than that previously reported [4], which may be associated with the selection of calculi location and the degree of intraoperative stone crushing. No residual calculi or calculi diameter ≤ 2 mm based on CT examination without clinical symptoms should be defined as stone-free status [11]. The stone-free rates at 3 months after surgery of tf-URS and FURS groups were lower than that of SMP group, but there was no significant difference.

Negative pressure suction can reduce the pressure over the renal pelvis and thereby reduce the occurrence of urosepsis [17]. Fever occurred more frequently in tf-URS group possibly because of the absence of intraoperative negative pressure suction and the small size of water outlet hole. As SN-V, SN-V1 and SN-V2 generation tf-URS equipped without a flexible ureteroscope sheath, the back-flow of lavage fluid decreased with high intrapelvic pressure [3]. When the intrapelvic pressure exceeds 30 mmHg, intrarenal backflow would occur and cause infection [18]. For patients with infection, it is advised to place double J stent two weeks before surgery when the infection is well controlled [19]. The three groups showed no significant difference in mucosa injury with only a few cases. The operation access diameter of tf-URS and FURS is F12-14. For patients with ureterostenosis, it is suggested to place double J stent in advance and perform second-time surgery [20]. The incidence of perirenal hematoma in SMP group was higher as artificial percutaneous renal access caused the damage to renal tissue. Perirenal hematoma in SMP group could be accompanied by lavage fluid exudation during Micro-PCNL, but the thickness of all perirenal hematomas was less than 2 cm, and they underwent self-absorption without arterial embolization. It is essential for SMP group to perform a precise puncture at the calyceal fornix. Besides, SMP access is too small to place the ureter forcep, so the blood clot cannot be removed during surgery with a blurry vision, especially when large bleeding volume exists. In this case, SMP has to be performed through other access.

However, some limitations still exists in this study. First, our study is retrospective in nature, requiring the use of prospective studies to validate the findings. Second, the long-term complications and efficacy were not assessed due to the insufficient follow-up time. Third, although all surgical procedures were conducted by a single surgeon who had been fully trained in these three surgical procedures, our results need to be tested by more surgeons in the future.

## Conclusions

Each of these three surgical techniques has advantages over the others. tf-URS is more advantageous in operation time, cost, and level of comfort. However, fever can occur more frequently after tf-URS which is not advised in patients with serious infection or ureterostenosis. SMP offers higher single stone crushing success rate, making it more suitable for calyceal or lower calyx calculi inaccessible by FURS, but it must be applied in combination with an advanced ultrasound instrument for precision puncture. By combining a negative pressure suction device, FURS is more suitable for upper ureteral calculi or pelvis calculi, providing high stone-free rate and patient comfort, but a disposable flexible ureteroscope can be very expensive.

## Data Availability

The data used and analyzed during this study are available from the corresponding author on reasonable request.

## Abbreviations

tf-URS: Sun’s tip-flexible semirigid ureterorenoscopy
SMP: super-mini percutaneous nephrolithotomy
FURS: flexible ureteroscopy
Micro-PCNL: micro-percutaneous nephrolithotomy
EAU: European Association of Urologists
AUA: American Urological Association
VAS: visual analog scale
SD: standard deviation
ANOVA: single factor analysis of variance
LSD: Fisher’s least significant difference
